# Preparation and In Vivo Pharmacokinetics of the Tongshu Suppository

**DOI:** 10.1155/2016/1691579

**Published:** 2016-08-16

**Authors:** Guoqiang Liu, Leilei Dong, Kuan Lu, Sisi Liu, Yingying Zheng

**Affiliations:** ^1^Department of Pharmacy, The Third Hospital of Hebei Medical University, Shijiazhuang 050051, China; ^2^The Third Hospital of Hebei Medical University, Shijiazhuang 050051, China

## Abstract

Astragalus polysaccharide (APS) (used for intestinal protection) was added to formulate the Tongshu suppository to improve the pharmacokinetics of Aceclofenac, which were assessed in New Zealand rabbits using an orthogonal experimental design. The single-agent Aceclofenac was taken as the control formulation. The concentration-time and drug release curves were drawn, and *T*_max_ (min), *C*_max_ (*μ*g·mL^−1^), AUC_0→*∞*_, and MRT were compared using a pharmacokinetic systems program. The formulated Tongshu suppository had moderate hardness, a smooth surface with uniform color, and theoretical drug-loading rate of 8%. Its release rate was in accordance with the drug preparation requirements. The concentration-time curves and drug release curves revealed that the maximum concentrations (*C*_max_) were 4.18 ± 1.03 *μ*g·mL^−1^ and 3.34 ± 0.41 *μ*g·mL^−1^ for the Tongshu and Aceclofenac suppositories, respectively, showing statistically insignificant difference, while the peak times were 34.87 ± 4.69 min and 34.76 ± 6.34 min, respectively, also showing statistically insignificant difference. Compared with the Aceclofenac suppository, the relative bioavailability of the Tongshu suppository was 104.4%, and the difference between them was statistically insignificant. In this experiment, the Tongshu suppository was prepared using the hot-melt method. In vivo pharmacokinetic studies confirmed it had higher bioavailability than the Aceclofenac suppository.

## 1. Background

Aceclofenac, 2-[2-[2-[(2,6-dichlorophenyl)amino]phenyl]-acetyl] oxyacetic acid, is a prostaglandin synthetase (cyclooxygenase) inhibitor which inhibits lipoxygenase and decreases prostaglandin production, thereby inhibiting the inflammatory process. Aceclofenac has been shown to have potent anti-inflammatory, analgesic, and antipyretic properties [1], which is indicated for acute and chronic treatment of the signs and symptoms of rheumatoid arthritis, osteoarthritis, ankylosing spondylitis, and scapulohumeral periarthritis [2]. Long-term treatment of intact cells with Aceclofenac has recently been shown to cause suppression of COX-2-dependent prostaglandin synthesis [3].

The usual dose of Aceclofenac is 100 mg twice a day; it is absorbed rapidly in its intact form when taken orally, and its analgesic effects begin within 30 min of ingestion [4]. Water-insolubility and gastrointestinal discomfort are two side effects, which widely influence the clinical use of Aceclofenac [5]. In order to improve its effective oral delivery, aceclofenac was mixed with various ratios of different solubilizers prepared using polyethylene glycol 400 (PEG-400) as the basic soft capsule ingredient [6].

*Astragalus* polysaccharides (APS) may enhance intestinal epithelial cell proliferation, migration, and differentiation in vitro by stimulating ODC gene expression and activity, and putrescine production, independent of TGF-*β*. Exogenous administration of APS may provide a new approach for modulating intestinal epithelial wound restitution in vivo [7].* Astragalus* polysaccharides promote the regeneration of damaged gastric mucosa, probably through their antioxidative mechanism [8]. In this study, the aceclofenac was mixed with various ratios of PEG 400 in order to develop an effective novel oral drug delivery system with accelerated absorption and reduced gastrointestinal discomfort, PEG-4000, glycerol and APS were used to make the Tongshu suppository, and their dissolution studies were performed. Furthermore, the pharmacokinetics of the Tongshu suppository and the aceclofenac suppository without APS were evaluated and compared in New Zealand rabbits.

## 2. Methods

### 2.1. Materials

This study was approved ethically by the Third Hospital of Hebei Medical University. The chemicals and reagents for this study were obtained as follows: Aceclofenac reference substance (China Pharmaceutical Biological Products Analysis Institute, Beijing, China), Aceclofenac drug substance (Xi'an Haixin Pharmaceutical Co., Ltd.),* Astragalus* polysaccharide (Xi'an Qingteng Bioscience Limited, Xi'an China), polyethylene glycol 400 (PEG400, Tianjin Yongda Chemical Reagent Co., Ltd., Tianjin, China), polyethylene glycol 4000 (PEG4000, Tianjin Guangfu Fine Chemical Research Institute, Tianjin, China), glycerol (Baishi Chemical Industry Co., Ltd., Tianjin, China), acetonitrile (chromatographic pure, American Grace Co., Shanghai, China), analytical reagents including monopotassium phosphate, caustic soda, and glacial acetic acid, and sodium acetate agents (Modern Instruments Co.).

### 2.2. Preparation of the Aceclofenac-Loaded Suppository with APS (Tongshu) or without APS (Control)

Aceclofenac was thoroughly blended with various solubilizers including PEG 400, PEG-4000, glycerol, and APS to formulate the Tongshu suppository. The control suppository was prepared with Aceclofenac thoroughly blended with PEG 400, PEG-4000, and glycerol without APS. These ingredients were rapidly transferred to a mold and frozen in the refrigerator for 30 min and then broken away from the mold after leveling. The detailed compositions of the Tongshu and control suppositories are given in Table 1.

### 2.3. Dissolution Test

Each Tongshu and control suppository was placed in a dissolution tester. The dissolution test was performed at 36.5°C using the paddle method at 100 rpm with 750 mL of distilled water as the dissolution medium [9]. At the predetermined interval, 2 mL aliquots of the medium were sampled and filtered. The filtrate was analyzed using the UV-Vis variable wavelength detector (Philips, Model PU8730) at 273 nm. The differences in dissolution rates of the drug from various preparations were compared using one-way analysis of variance (ANOVA). The significance between the means of different formulations was then compared by the multiple range method of least significant difference.

### 2.4. Pharmacokinetic Study

#### 2.4.1. Animals and Treatments

This experiment was designed as a two-cycle crossover trial for two agents [10, 11], with a washing period of one week. Before the experiment, six New Zealand rabbits were randomized into two groups and were fasting for 12 h. The first group was then given one piece of the Tongshu suppository (containing 100 mg Aceclofenac) and the second group was given the Aceclofenac suppository (100 mg); the intact suppository was inserted about 1 cm into the rabbit anus, followed by compression for about 15 min to avoid excretion. After that, 1 mL of blood was drawn from the ear vein at 0.25, 0.5, 1, 1.5, 2, 2.5, 3, 4, 6, 8, and 10 h after administration and placed into 2 mL Eppendorf tubes which were pretreated with heparin and centrifuged at 3000 r/min for 10 min. The upper plasma layer was then collected and cryopreserved at −20°C for subsequent experiments. In the crossover trials in the second week, the Tongshu suppository group in the last week was changed to the Aceclofenac suppository group, while the Aceclofenac suppository group was changed to the Tongshu suppository group, using abovementioned steps.

#### 2.4.2. Administration and Blood-Collection

Processing the plasma samples, 0.5 mL blank plasma was accurately weighted and placed into a 2 mL EP tube. After that, 500 *μ*L of acetonitrile was added, followed by vortex mixing for 3 min and centrifugation at 10000 r·min^−1^ for 10 min in a high speed centrifuge. Then the upper organic phase was collected into a 1 mL syringe and filtered with a 0.45 *μ*m filtration membrane, from which 15 *μ*L was used for LC analysis.

#### 2.4.3. Calculation of Pharmacokinetic Parameters

All pharmacokinetic parameters were determined by noncompartmental analysis. AUC was calculated using the linear trapezoidal method. *C*_max_ (the highest drug concentration measured) and *T*_max_ (the time to reach the highest concentration) were directly read from the concentration/time plots. The maximum concentrations (*C*_max_) were 4.18 ± 1.03 *μ*g/mL and 3.34 ± 0.41 *μ*g/mL for the Tongshu and suppositories, respectively, while the peak times were 34.87 ± 4.69 min and 34.76 ± 6.34 min, respectively.

### 2.5. Statistic Analysis

The concentrations measured at various time points were fitted using 3p97 software. The mean values of plasma-drug concentrations at each sampling point were taken as the primary and reference data, to which each individual value was fitted. AIC was considered as a judged index to determine the compartmental model and weight for mandatory fitting of the other individual data using the principle of majority. This study aimed to establish a preparation method of self-made suppository and do preliminary research on in vivo pharmacokinetics. Due to rectal administration itself, it is not appropriate to use the existing data of the conventional tablets of oral administration as reference in the in vivo studies of suppository. This study aimed to establish a preparation method of self-made suppository and do preliminary research on in vivo pharmacokinetics. Due to rectal administration itself, it is not appropriate to use the existing data of the conventional tablets of oral administration as reference in the in vivo studies of suppository. In this study, the reference suppository is also self-made and the experimental conditions and mode of administration and so forth are also the same as the study suppository, so the conclusions are more comparable.

## 3. Results and Discussion

### 3.1. Analyses of the Drug Release Curves of the Tongshu Suppository

**Figure 1:**
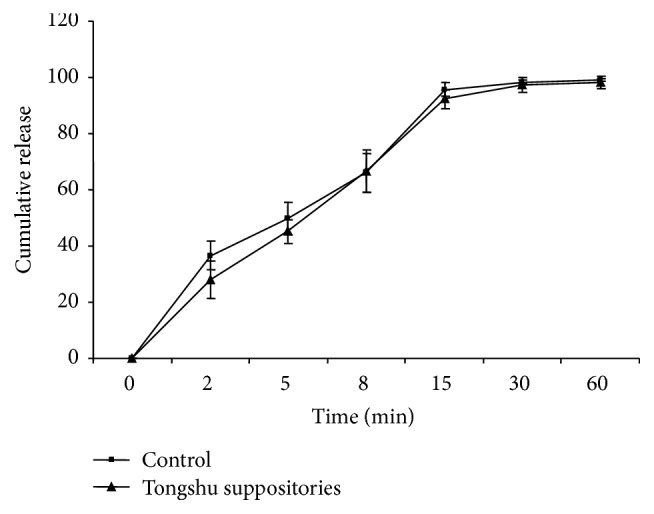
Drug release curves of the Tongshu and Aceclofenac suppositories.

The drug release curve shown in Figure 1 indicates that the Tongshu suppository is a quick release suppository. Upon introduction of the APS additive, the early dissolution speed of Aceclofenac decreased; however, its dissolution rate increased significantly with continuous release of APS into the phosphate buffer.

### 3.2. Analyses of the Concentration-Time Curves of the Tongshu Suppository

As can be seen from the concentration-time curves of the Tongshu suppository, it was quickly absorbed into the blood, showed fast elimination, and did not exhibit significant differences in these parameters compared with the concentration-time curves of the Aceclofenac suppository. Also, the drug release parameters observed in vitro were similar for both suppositories.

### 3.3. Pharmacokinetic Analyses of the Tongshu Suppository

Pharmacokinetic analyses were performed using 3p97 software, and the results revealed that the Tongshu suppository mainly presented as one compartment model, suggesting that it could be promptly absorbed into the blood through venae intestines after rectal administration. This observation was also confirmed by the data processing results, where the *T*_max_ was 34.87 min in the Tongshu suppository group, which was longer than the peak time of gavage of the Aceclofenac suspension alone. And *C*_max_ was 4.57 ± 1.09 *μ*g·mL^−1^ in the Tongshu suppository group, which was longer than the peak time of gavage of the Aceclofenac suspension alone. The *K*_el_ was 0.022875 ± 0.007 min^−1^, which was higher than the peak time of gavage of the Aceclofenac suspension alone. Otherwise, AUC_0→*∞*_ is 496.52 ± 2.4 *μ*g·min·mL^−1^ in the Tongshu suppository group and MRTis 171.395 ± 3.08 min in the Tongshu suppository group, where both significantly decreased compared with that in control group (Table 2).

Aceclofenac is a tendentious COX-2 inhibitor mainly used in symptomatic treatment of osteoarthritis, rheumatoid and juvenile rheumatoid arthritis, and ankylosing spondylitis. It is insoluble in water, easily soluble in acetone and dimethylformamide, and also soluble in methanol and ethanol. It is prone to causing side effects on the gastrointestinal tract after oral administration; in addition, it is also a COX inhibitor, which tends to aggravate its stimulating effects on the gastrointestinal tract. This study aimed to explore the ways of reducing the side effects of Aceclofenac and improving its safety and compliance by changing the route of its administration and adding adjuvant APS to the formulation.

In drug release, the APS additive tended to decrease the early dissolution speed of Aceclofenac, but this effect was not statistically significant. This finding might be due to dispersion of Aceclofenac molecules in the polyethylene glycol matrix and their quick release into the medium during dissolution. APS is a water-soluble medium, with certain affinity for the polyethylene glycol matrix. However, it takes some time to achieve the dynamic balance between the dissolved medium and the matrix. With continuous release of APS into the phosphate buffer, the dissolution rate increased at certain times. Because APS and Aceclofenac are mixed and added to the matrix, Aceclofenac tends to achieve quick dissolution with the release of APS, resulting in a dissolution rate comparable to that of the Aceclofenac suppository.

The mobile phase was screened during HPLC analysis of Aceclofenac. Methanol was selected as the organic phase in the early stage [12, 13]. However, unsatisfactory results were obtained since absorption occurred at 273 nm in the blank suppository. This might be due to the fact that the samples were not thoroughly cleaned by the washing solution (50% methanol) or that the instrument itself was not optimally cleaned. Furthermore, methanol itself has a high viscosity, leading to failure in achieving thorough rinsing even using the maximum aspiration speed of the needle washing machine. Therefore, acetonitrile was selected in our experiment [1, 12, 14] and the adjusted mobile phase consisted of acetonitrile-sodium acetate. Initially, the chosen ratio was 60 : 40, giving rise to a peak at about 5 min and nonideal separation. Then the ratio of the organic phase was decreased; that is, a 45 : 55 acetonitrile-sodium acetate ratio was used, which resulted in good separation. The accuracy, precision, and stability of this method were in accordance with the experimental requirements and no interference from adjuvant drugs was observed.

In terms of blood sample management, acetonitrile was selected as the solvent for the albumin precipitant of the plasma samples. In order to ensure the accuracy of blood concentration monitoring, we did not adopt a redissolution method. Furthermore, this method had poor repeatability and yielded impure peaks. Therefore, the filtered samples were directly drawn after the deposition process, which was confirmed to be methodologically appropriate for investigation of the in vivo pharmacokinetics of this preparation [15, 16].

In terms of in vivo analyses, the concentration-time curve and bioavailability were not statistically significantly different between the Aceclofenac and Tongshu suppositories. Compared with the oral formulations of the same products, the Tongshu suppository was unlikely to cause stimulation of the gastrointestinal tract due to nonoral administration. Furthermore, the APS additive had a certain protective effect on intestinal mucosa.

According to the literature, the Tongshu suppository showed significantly improved peak time and AUC_0→*∞*_ in comparison with domestic and foreign Aceclofenac oral formulations. In addition, it also showed improved bioavailability in comparison with the homemade Aceclofenac suppository tested in this experiment. However, Aceclofenac had a lower in vivo peak concentration and shorter MRT, presenting with quick elimination in vivo [17–19]. Due to small sample size, the data from this study showed a high degree of dispersion due to individualized differences among animals, leading to poor representation. Therefore, increasing the sample size of in vivo pharmacokinetic studies is necessary, in order to obtain more useful experimental data.

**Figure 2:**
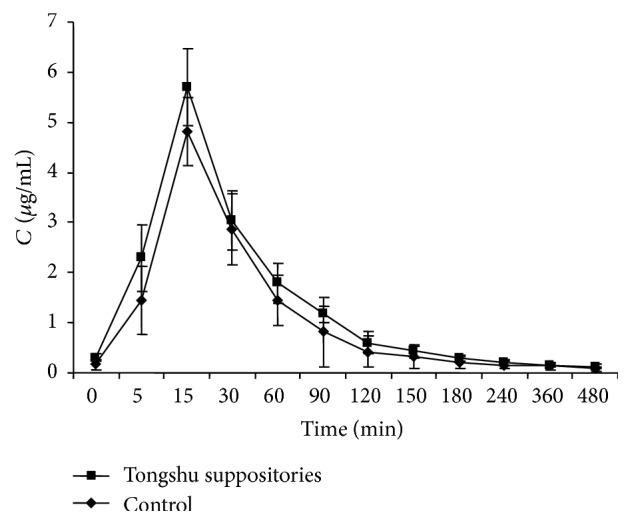
Concentration-time curves of the Tongshu and Aceclofenac suppositories.

This study points to several important advantages of the Tongshu suppository which warrant further investigation. First, the drug release, absorption, and bioavailability of the active ingredient from the Tongshu suppository were comparable to that observed for the Aceclofenac suppository as indicated in Figures 1 and 2. Second, the advantage of the Tongshu suppository according to the results of pharmacokinetic studies is that it is quickly absorbed into the blood due to rectal administration, as opposed to Aceclofenac oral formulations studied earlier [20]. The results from Table 2 indicate that *T*_max_ of the Tongshu suppository was 2.31 min longer in comparison to the *T*_max_ of the Aceclofenac suppository. Third, although the APS additive from the Tongshu suppository slightly decreased the early dissolution speed of Aceclofenac, it also offered protective effects on gastrointestinal mucosa. In conclusion, formulated Tongshu suppository is characterized by fast absorption and lack of stimulative effects on the digestive system. However, its antiarthritic effects and protective effects on intestinal mucosa require further investigation. Moreover, its metabolism and removal in vivo have not been studied yet and would make for an interesting topic of future studies.

## 4. Conclusions

The formulation and preparation of Tongshu suppository were simple and feasible, with good reproducibility. The methods have established for the determination of content and dissolution. The concentration of plasma-drug concentration determination method was established, for the study of the preparation of quality guarantee. In vivo pharmacokinetic studies confirmed it had higher bioavailability than the Aceclofenac suppository.

## Figures and Tables

**Table 1:** Preparation of the Tongshu and Aceclofenac suppositories.

	Tongshu	Aceclofenac
PEG400	0.49	0.48
PEG4000	0.49	0.48
Glycerol	0.12	0.14
APS	0.1	0
Aceclofenac suppository	0.1	0.1

**Table 2:** Pharmacokinetic analyses of Tongshu and Aceclofenac suppositories. The data are expressed as mean ± SD.

Parameter	Tongshu suppository	Aceclofenac suppository
	Mean ± SD	Mean ± SD
*T*_max_ (min)	32.6 ± 3.2	30.29 ± 2.5
*C*_max_ (*μ*g·mL^−1^)	4.57 ± 1.09	3.14 ± 0.5
*K*_el_ (min^−1^)	0.022875 ± 0.007	0.01885 ± 0.009
*K*_a_ (min^−1^)	0.0553 ± 0.02	0.0573 ± 0.01
AUC_0→*∞*_ (*μ*g·min·mL^−1^)	496.52 ± 2.4	580.75 ± 234.5
MRT (min)	171.395 ± 3.08	345.12 ± 60.6
